# Glucosylceramide Synthase, a Key Enzyme in Sphingolipid Metabolism, Regulates Expression of Genes Accounting for Cancer Drug Resistance

**DOI:** 10.3390/ijms26115112

**Published:** 2025-05-26

**Authors:** Md Saqline Mostaq, Lin Kang, Gauri A. Patwardhan, Yunfeng Zhao, Runhua Shi, Yong-Yu Liu

**Affiliations:** 1 School of Basic Pharmaceutical and Toxicological Sciences, University of Louisiana at Monroe, Monroe, LA 71201, USA; mostaqms@warhawks.ulm.edu (M.S.M.); gaurianandpatwardhan@gmail.com (G.A.P.); 2 Department of Biomedical Affairs and Research, Edward Via College of Osteopathic Medicine, Monroe, LA 71203, USA; lkang@ulm.vcom.edu; 3 Department of Biomedical Sciences and Pathobiology, Virginia Tech, VA-MD Regional College of Veterinary Medicine, Blacksburg, VA 24060, USA; 4 Department of Pharmacology, Toxicology and Neuroscience, Louisiana State University Health Sciences Center, Shreveport, LA 71103, USA; yunfeng.zhao@lsuhs.edu; 5 Department of Internal Medicine, Louisiana State University Health Sciences Center, Shreveport, LA 71103, USA; runhua.shi@lsuhs.edu

**Keywords:** microarray, gene mutation, p53 tumor suppressor, glucosylceramide synthase, apoptosis, dactinomycin, ovarian cancer

## Abstract

Emergent cancer drug resistance and further metastasis can mainly be attributed to altered expression levels and functional activities of multiple genes of cancer cells under chemotherapy. In response to challenge with anticancer drugs, enhanced ceramide glycosylation catalyzed by glucosylceramide synthase (GCS) confers drug resistance and enrichment with cancer stem cells. p53 mutations, which gain function in tumor progression, are prevalently extant in ovarian cancers. Via integrated gene expression assessments, we characterized GCS-responsive genes in ovarian cancer cells treated with dactinomycin. NCI/ADR-RES cells dominantly expressed a p53 mutant (7 aa deleted in exon-5) and displayed anti-apoptosis; however, silencing GCS expression rendered these cells sensitive to dactinomycin-induced apoptosis. Microarray analyses of NCI/ADR-RES and its GCS transfected sublines found that elevated GCS expression or ceramide glycosylation was associated with altered expression of 41 genes, notably coding for ABCB1, FGF2, ALDH1A3, apolipoprotein E, laminin 2, chemokine ligands, and IL6, with cellular resistance to induced apoptosis and enrichment with cancer stem cells, promoting cancer progression. These findings were further corroborated through integrated genomic analyses of ovarian cancer from The Cancer Genome Atlas (TCGA) and cancer resistance to platinum-based chemotherapy. Altogether, our present study indicates that altered ceramide glycosylation can modulate expression of these GCS-responsive genes and alter cancer cell attributes under chemotherapy.

## 1. Introduction

Cancer drug resistance causes treatment failure in over half of cancer patients, and increases incidence of metastases in multiple organs. In response to cellular stress under chemotherapy, cancer cells mainly overexpress groups of genes that function in protecting cancer cells via strengthening drug resistance and tumor metastability. Among endogenous biomolecules and pathways characterized as effectors of cancer drug resistance, glucosylceramide synthase (GCS)-catalyzed ceramide glycosylation directly decreases cellular pro-apoptotic ceramide levels and increases anti-apoptotic glucosylceramide (GlcCer) levels [1,2]. GCS is a rate-limiting enzyme of serial glycosylation for biosynthesis of glycosphingolipids (GSLs) [3,4]. GCS-catalyzed ceramide glycosylation occurs in the Golgi apparatus of cells, thereby providing GlcCer as a precursor for producing various GSLs. In mammalian cells, GSLs and other constituents, including sphingolipids, sterols and membrane-associated proteins, comprise the GSL-enriched microdomain (GEM), a unique lipid raft marked with flotillins (Flot-1 and Flot-2) [5,6]. By interactions or associations with membrane proteins and signal transducers in GEMs, GSLs actively modulate the functional effects of proteins, including Src family kinases [6,7]. Previous reports indicate that GSLs play essential roles in modulating the transcription of several genes via cSrc and β-catenin signaling pathways [8,9]. Upon challenge with various anticancer drugs, increased Cer glycosylation and cellular GSLs can up-regulate the expression of particular genes, including multidrug resistance 1 (MDR1, also known as ABCB1), fibroblast growth factor 2 (FGF2), and even p53 mutants, protecting cancer cells against chemotherapy [8,9,10].

Drug resistance is prevalent in solid cancers such as ovarian cancer. Ovarian cancer is the fifth-leading cause of cancer death among women in the United States; 21,410 new cases and 13,770 deaths were estimated to have occurred in 2021 [11]. Most deaths (~70%) are of patients presenting with advanced-stage and high-grade serous ovarian cancer (HGS-OvCa), almost all carrying gene mutations of *TP53* (96%) and exhibiting poor response to chemotherapy [12,13]. After aggressive surgery followed by chemotherapy, cancer recurs in approximately 25% of patients within six months, most often is treatment-resistant, and the overall five-year survival probability is only 31% [14]. Wild-type *p53* (wt *p53*) protein, encoded by the gene *TP53,* acts as a potent transcription factor that promotes the expression of p53 target genes, including p21, Bax, Puma, and others, thereby effectively executing cell proliferation arrest or apoptosis in response to genotoxic stress [15]. p53 mutants, about 75% of which are missense, enact oncogenic effects and are causative of cancer drug resistance and cancer progression [16,17,18].

Integrated analyses of cancer cells with clinical datasets can enable systemic assessments that may help to elucidate key molecular contributors to biological processes, including cancer drug resistance and metastasis [19,20]. To understand how cancer cells carrying p53 mutants respond to anticancer drugs so as to gain resistance, we investigated whether the introduction of GCS confers cancer drug resistance, and further, identified GCS-responsive genes that contribute to anti-apoptosis in p53-mutant-carrying cancer cells.

## 2. Results

### 2.1. Silencing GCS Expression Sensitized NCI-ADR-RES Cells to Dactinomycin-Induced Apoptosis

**Figure 1:**
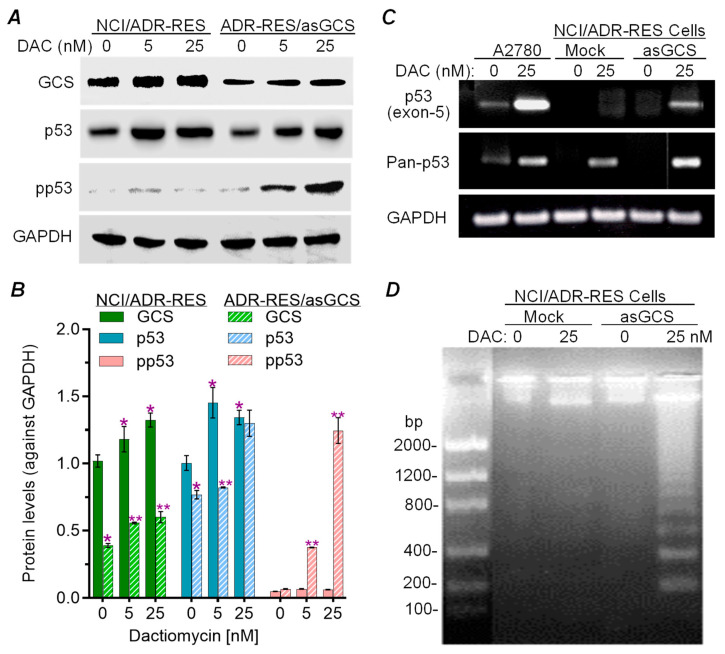
GCS regulates p53 expression and cell response to dactinomycin-induced apoptosis. Cells of NCI/ADR-RES and ADR-RES/asGCS lines were treated with dactinomycin (DAC) for 48 h. (**A**)**,** Western blotting of GCS and p53 proteins. Equal amounts of soluble proteins (50 μg/lane) of cells were resolved by SDS-PAGE, and the immunoblots were against corresponding antibodies (1:500 or 1:1000). pp53, phosphorylated p53 (Ser15). (**B**), Effects of GCS on p53 protein expression. Relative levels of proteins were presented as density values of each protein, normalized against GAPDH, from different blot analyses. *, *p* < 0.001 compared to NCI-ADR-RES cells treated with vehicle; ** *p* < 0.001 compared to NCI-ADR-RES cells treated with DAC. (**C**), pre-mRNA of p53. Equal amounts total RNA (100 ng/each) were amplified using PCR with corresponding primers after reverse reaction, and resolved by 1% agarose gel electrophoresis. Pan-p53, undistinctive p53 RNA. (**D**), Apoptotic DNA fragmentations. Equal amounts of DNA extracts (2 μg/lane) were resolved by 2% agarose gel electrophoresis and visualized with ethidium bromide (EB) staining. Mock, NCI/ADR-RES cells; asGCS, ADR-RES/asGCS cells.

Previous studies showed that introduction of GCS gene knock-in conferred cancer cell resistance to commonly used chemotherapeutic agents, and conversely, that suppressing GCS activity either directly with GCS inhibitors, or by silencing its expression, sensitized cancer cells to these anticancer agents [1,21,22,23,24]. Parental cells of NCI/ADR-RES carry a p53 mutation, with expression dominance of mutant p53 mRNA having a 21 bp deletion in a segment coding for the DNA-binding domain (exon 5 between codons 126–132). These cells display multidrug resistance [25,26,27,28]. With antisense knock-down (asGCS), the resultant ADR-RES/asGCS cells expressed significantly lower levels of GCS protein, compared with ADR-RES/GCS cells, upon parallel treatments with dactinomycin (DAC, 5 and 25 nM) (Figure 1A). Concordantly, levels of phosphorylated p53 (pp53) were significantly increased in ADR/RES/asGCS cells upon DAC treatments at 5 and 25 nM (Figure 1A), but not in ADR-RES/mock cells. asGCS transfection restored levels of intact exon-5 mRNA in ADR-RES/asGCS cells with DAC treatments (25 nM for 48 h) (Figure 1B). Further, we found that DAC treatments induced apoptosis in ADR-RES/asGCS cells but not mock-transfected cells, as indicated by DNA fragmentation (Figure 1C). These results indicate that GCS, a rate-limiting enzyme in ceramide glycosylation, can substantially affect the DNA-damage stress response of cancer cells, possibly via modulating the expression of other genes (vide subra) in response to treatment with DAC.

### 2.2. GCS Is Involved in Regulating Expression of Genes Protecting Cancer Cells

**Figure 2:**
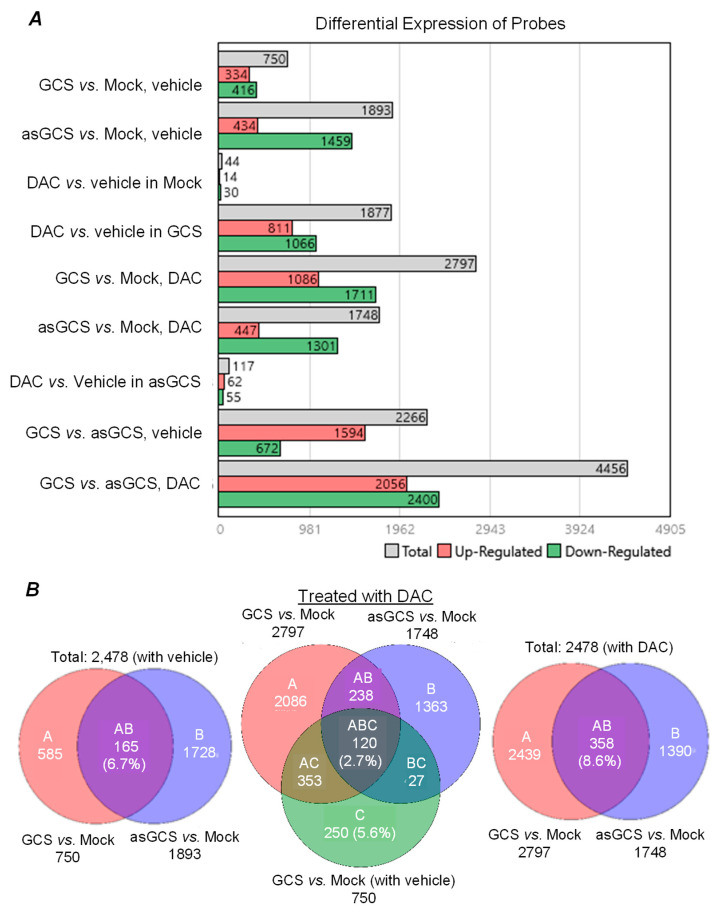
GCS is involved in modulating gene expression of ovarian cancer cells in response to dactinomycin treatments. Cells of NCI/ADR-RES/Mock (Mock), NCI/ADR-RES/GCS (GCS), and NCI/ADR-RES/asGCS (asGCS) ovarian cancer cell lines were treated with 25 nM dactinomycin (DAC) in 5% FBS for 24 h. The mRNA levels of genes were analyzed by using Affymetrix GeneChip^®^ Human Genome U133 Plus 2.0 and Transcriptome Analysis Console (TAC 4.0). (**A**), differential gene-expression profiles of NCI/ADR-RES cells in response to DAC treatments. Differential expression of probed genes was identified as those having transcript levels 2-fold higher (up-regulated) or 2-fold lower (down-regulated) for the cells with treatment than those of comparator cells. (**B**), Overlaps of gene profiles of NCI/ADR-RES cells with treatments. GCS-responsive genes (overlap AB areas) are identified as those exhibiting their expression levels up-regulated in ADR-RES/GCS cells versus down-regulated in ADR/RES/asGCS cells (vice versa) treated with vehicle (left) or DAC (right), respectively.

With Affymetrix GeneChip^®^, we assessed differential gene-expression profiles of NCI/ADR-RES/mock (Mock) cells, and cells of the parallel GCS knock-in and knock-down sublines ADR-RES/GCS (GCS) and ADR-RES/asGCS (asGCS), upon treatment with DAC (25 nM for 24 h). Analyses with TAC indicated that the transfection of asGCS into ADR-Res/asGCS cells caused differential expression of a substantially greater number of probed genes (1143 additional probed genes: up-regulated 100, down-regulated 1043 beyond the arbitrarily set twofold threshold) than comparable GCS knock-in of ADR-RES/GCS cells (Figure 2A). As anticipated, for NCI-ADR-RES/mock cells, we observed differential expression of only 44 more probed genes (14 up-regulated, down-regulated) upon DAC treatment, as compared to vehicle. By comparison to ADR-RES/mock treated with DAC, GCS knock-in (ADR-RES/GCS cells) exhibited a markedly greater number of differentially expressed genes than the similar comparison for GCS knock-down (ADR-RES/asGCS), 1051 more in total (639 more genes differentially up-regulated, 410 more genes differentially down-regulated) (Figure 2A). In direct comparison with ADR-RES/asGCS, ADR-RES/GCS cells differentially expressed 4456 more probed genes (2056 up-regulated, 2400 down-regulated) in response to DAC treatments (Figure 2A). This evidence indicates that DNA-damage stress caused by DAC, in the context of enhanced ceramide glycosylation by GCS, substantially impacts compensatory gene expression changes in cancer cells as a response to treatments so as enable cell survival.

We further compared these differential gene-expression profiles, localizing for probe overlap (AB overlap sets) between ADR-RES/GCS cells and ADR/RES/asGCS cells. These comparisons identified 358 overlapping probed genes in cells treated with DAC (Figure 2B, right) as compared to only 186 genes in cells treated with vehicle (Figure 2B, left). These probed genes (identified from the AB overlap sets) are presumably those responsive to elevated GCS, as the expression levels of these genes were up-regulated with GCS transfection, whereas they were down-regulated with asGCS transfection, or vice versa. Further, we identified 120 probed genes (overlap ABC area, Figure 2B center) wherein gene expression levels upon DAC treatment as compared to vehicle were not only up-regulated in GCS knock-in cells, but were also down-regulated in asGCS knock-down cells (and vice versa).

### 2.3. GCS-Responsive Genes Are Involved in Modulating Cancer Drug Resistance and Metastasis

**Figure 3:**
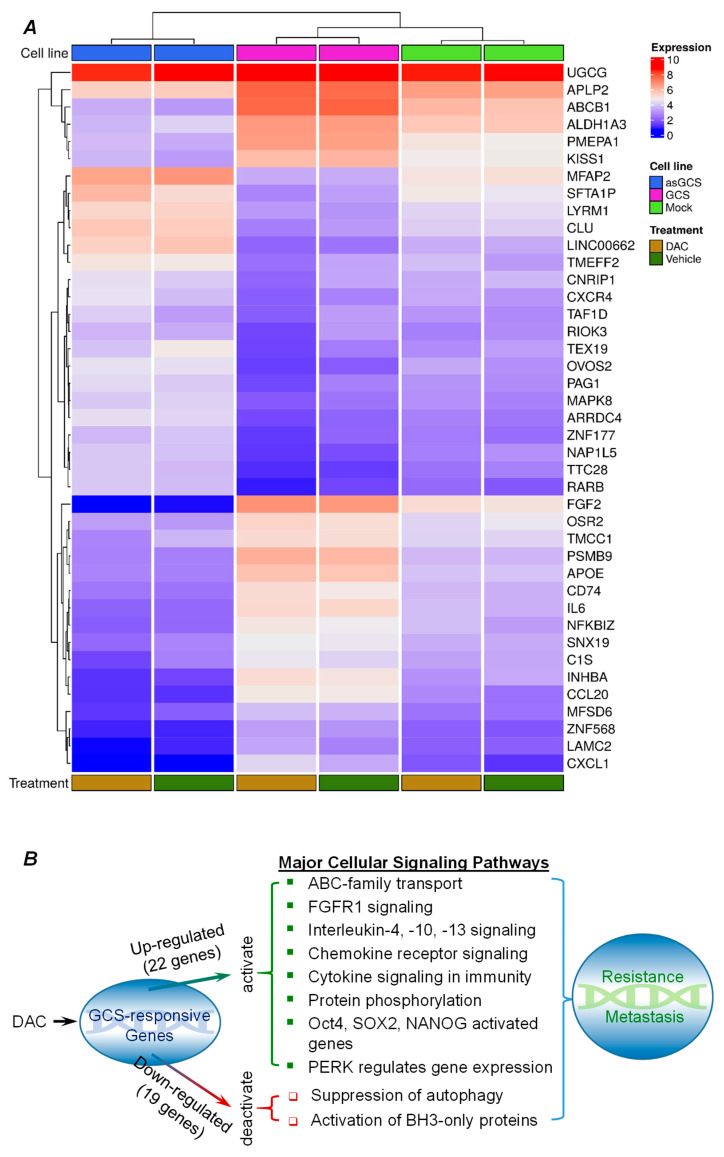
GCS-responsive genes of ovarian cancer cells in response to DAC treatments. (**A**), Heatmap of 41 genes with ratios indicating fold change between 0.2 and 9 (arrays labeled) among six experimental conditions. Columns represent array experiments and rows represent genes. The direction of expression ratios is indicated by red and blue, and the magnitude of the ratios is reflected by the degree of color saturation (see color scale). GCS, ADR-RES/GCS cells; Mock, ADR-RES/Mock cells; asGCS, ADR-RES/asGCS cells; DAC, 25 nM dactinomycin treatment for 24 h. (**B**), Pathways in which these GCS-responsive genes are involved.

Among these integrally identified probed genes (overlap ABC area, Figure 2B) of NCI/ADR-RES cell lines altered upon DAC treatment, we characterized 41 genes, for which transcript level changes tightly correlated with GCS (Figure 3A, Table 1). Under DAC treatments, GCS transfection caused differential up-regulation of genes, including the following 22: CXCL8, PSMB9, CXCL1, INHBA, CCL20, APOE, ABCB1, FGF2, PMEPA1, CD74, IL6, MFSD6, LAMC2, C1S, APLP2, SNX19, ZNF568, NFKBIZ, OSR2, KISS1, ALDH1A3, and TMCC1. Conversely, asGCS transfection down-regulated the expression levels of these same 22 genes (Table 2). Another 19 genes (including GALC, RIOK3, ARRDC4, TAF1D, MAPK8, LYRM1, ZNF177, TTC28-AS1, CNRIP1, NAP1L5, TEX19, RARB, LINC00662, CXCR4, PAG1, CLU, TMEFF2, MFAP2, OVOS2, and SFTA1P) were down-regulated by GCS transfection vs. up-regulated by asGCS transfection in NCI/ADR-RES cells treated with DAC (Table 2).

To explore how these GCS-responsive genes might alter cancer cells so as to bestow drug resistance and metastasis, we identified via REACTOME pathways within which the protein products of these GCS-responsive genes participate. The results show that among GCS-responsive genes, GCS up-regulated genes (*n* = 22) may activate various survival-promoting cellular signaling pathways, notably including those involving the following: (1) ABC-family transporters (ABCB1); (2) FGF receptor signaling (FGF2); (3) interleukin-4, -10, and -13 signaling (CCL20, CXCL8, CXCL1, IL6, FGF2, PSMB9, APLP2, and APOE); (4) chemokine receptors (CCL20, CXCL1, CD74, and CXCL8); (5) cytokine signaling in immunity (CCL20, CXCL1, CXCL8, FGF2, PSMB9, and IL6); (6) post-translational protein phosphorylation (APLP2, APOE, and IL6); (7) peptide ligand-binding receptors (CCL20, CXCL8, CXCL1, KISS1, and CD74); (8) PERK (EIF2AK3)-mediated gene expression (CXCL8 and APOE); and (9) the Oct4, SOX, NANOG activated genes related to proliferation (FGF2) (Figure 3B). On the other hand, pronounced GCS down-regulated genes (n = 19) were associated with survival-promoting deactivation of selected cellular signaling pathways, notably including those involved with the following: (1) suppression of autophagy (MAPK8); and (2) activation of BH3-only proteins (MAPK8) (Figure 3B).

### 2.4. GCS-Responsive Genes Are Highly Expressed in Ovarian Cancer and Correlated with Platinum-Resistance

**Figure 4:**
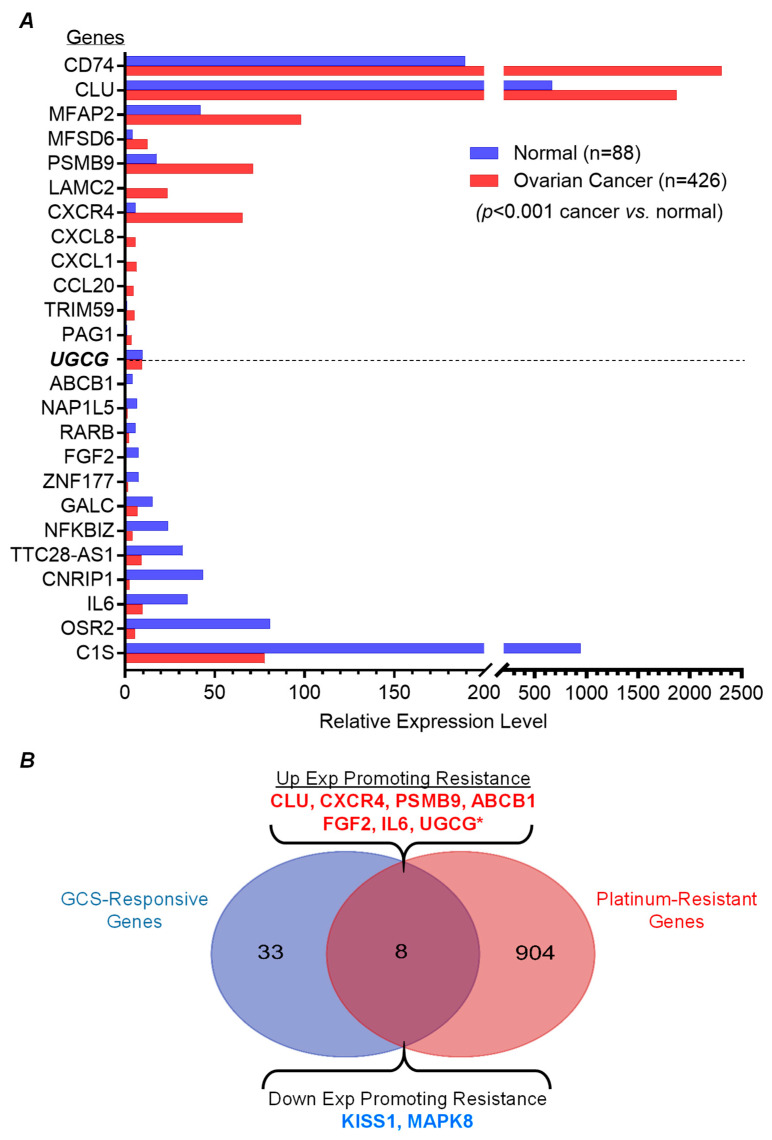
GCS-responsive genes are correlated with ovarian cancer and platinum-resistance of cancers. (**A**), GCS-responsive genes (24/41) are highly expressed correlatively in ovarian cancers of patients (*p* > 0.001 compared to normal ovarian tissues). (**B**), GCS-responsive genes (8/41) are highly correlated with platinum-resistance of cancers. *, UGCG encoding GCS.

We further evaluated GCS-responsive genes in ovarian cancer patients via the National Cancer Institute GDC Data Portal (https://portal.gdc.cancer.gov/; accessed on 13 June 2024) and explored their expression in ovarian cancer samples from TCGA and GTEx data via GEPIA (http://gepia.cancer-pku.cn/; accessed on 13 June 2024) [29]. Among 426 cases of ovarian cancer collected, it was found that 21 of the identified GCS-responsive genes (21/41) were differentially expressed, significantly so (*p* < 0.001), in ovarian cancer (Figure 4A) (Table 3). Among these, expression levels for ten genes (including CD74, CLU, MFAP2, MFSD6, PSMB9, LAMC2, CXCR4, CXCL1, CCL20, and PAG1) were significantly increased in ovarian cancer (*p* < 0.001, Figure 4A). For another 11 of the identified genes (including ABCB1, NAP1L5, RARB, FGF2, ZNF177, NFKBIZ, TTC28-AS1, CNRIP1, IL6, OSR2, and C1S), expression levels were significantly decreased in ovarian cancer cases (Figure 4A).

Chemotherapy for ovarian cancer usually entails a combination of a *platinum compound* (cisplatin or carboplatin) and a *taxane* (paclitaxel or docetaxel). Initial response rates are 60–80%, but eventually the majority of patients become platinum-resistant (response rates < 15%) with subsequent relapse [30,31]. Previous studies showed that overexpression of GCS resulted in platinum-resistance of cancer cells and xenograft tumors [32,33,34]. Recently, Huang et al. provided a comprehensive overview, based on databases of genomics and proteomics over the last 30 years, indicating that more than 900 genes having up-regulated expression (including *UGCG*, coding for GCS) were associated with platinum-resistance in cancers [35]. After mining these databases (http://ptrc-ddr.cptac.data-view.org/#/, accessed on 13 June 2024), we found that some of identified GCS-responsive genes are among those known to be strongly associated with platinum-resistance in cancer [35]. To wit, 8 of the 41 GCS-responsive genes (8/41) are also among those genes reported to correlate with platinum-resistance (Figure 4B). Interestingly, for six genes (KISS1, CXCR4, PSMB9, ABCB1, FGF2, and IL6) found to be up-regulated along with GCS, their overexpression pursuant to platinum-agent treatments in patients promoted resistance to those same agents in cancers (Figure 4B). Two genes (CLU, MAPK8) for which expression was suppressed upon GCS knock-down were also reported to promote cancer platinum-resistance (Figure 4B). This evidence clearly supports the contention that GCS-responsive gene expression changes serve to promote drug resistance of cancer cells.

## 3. Discussion

**Figure 5:**
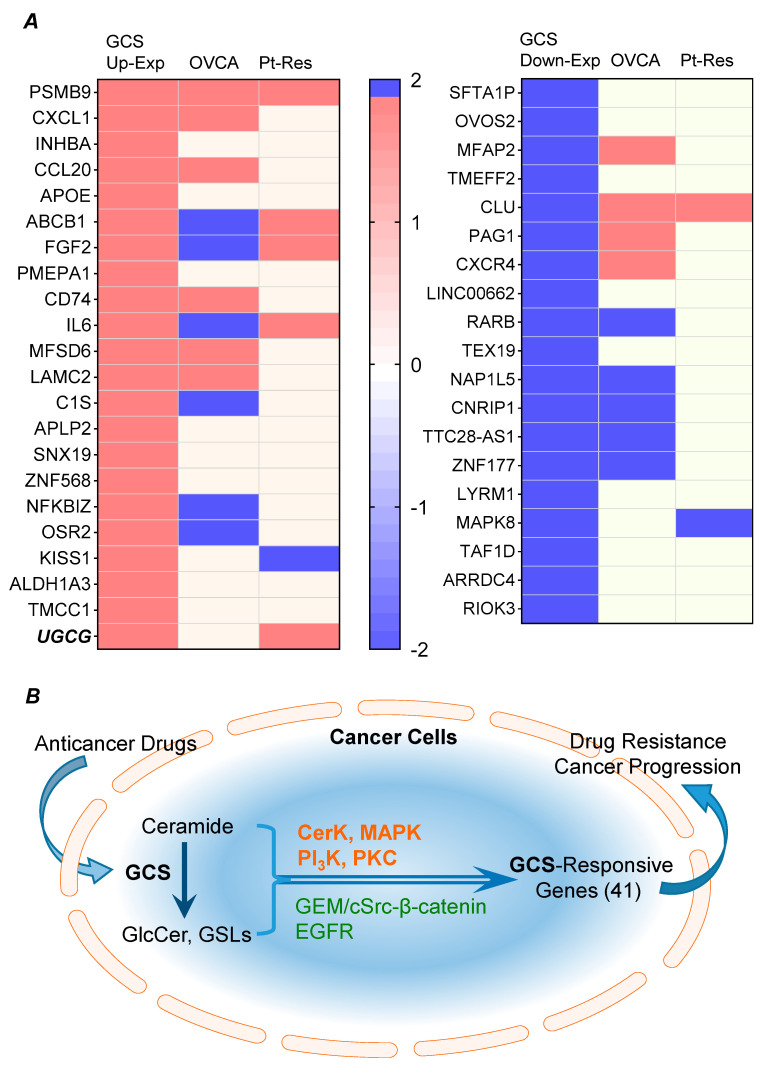
GCS-responsive genes relay drug resistance in cancer under treatments. (**A**), The correlation of GCS-responsive genes with genes associated with ovarian cancer and cancer resistance to platinum-based chemotherapy. (**B**), Ceramide glycosylation catalyzed by GCS modulates gene expression, leading to drug resistance and progression. Aberrant glycosphingolipid presences (GSLs, including glucosylceramide (GlcCer), globo-series, ganglio-series) in GSL-enriched membrane microdomains (GEMs) activate cSrc signaling (or EGFR) to enhance expression of GCS-responsive genes (i.a., *ABCB1*, *FGF2*, *IL6*). Conversely, reduced ceramide in GEMs or cytoplasm inactivates ceramide kinase (CerK) or MAPK, PI_3_K, and PKC to repress gene expression. Aberrant levels of globo-GSLs or ceramide also can alter pre-mRNA splicing to promote expression of mutant protein of p53 or of anti-apoptotic Bcl-x and caspase-9.

In response to stress, cancer cells have been observed to overexpress GCS to enhance ceramide glycosylation, resulting in cell resistance to treatments with anticancer drugs [23,24,25,36]. Previous studies showed that aberrantly elevated expression of GCS in turn promotes the overexpression of ABCB1, FGF2, and IL6 in cancer cells [8,9,34,37]. For the first time, this study systemically identified the 41 GCS-responsive genes that are responsible for drug resistance in ovarian cancer cells carrying a *TP53* mutation. Overexpression of GCS in turn up-regulated expression of 22 genes (PSMB9, CXCL1, INHBA, CCL20, APOE, ABCB1, FGF2, PMEPA1, CD74, IL6, MFSD6, LAMC2, C1S, APLP2, SNX19, ZNF568, NFKBIZ, OSR2, LOC643201, KISS1, ALDH1A3, and TMCC1). Interestingly, six genes among these (ABCB1, FGF2, CXCR4, IL6, PSMB9, and CLU), as well as UGCG, are known to be highly associated with cancer resistance to platinum-based chemotherapy [35]. Overexpression ABCB1, FGF2, CXCR4, and IL6 portends aggressive ovarian cancer behaviors and poor prognoses. Several studies indicate that overexpression of ABCB1 (also named MDR1) correlates with drug resistance in ovarian cancer [8,38,39]. Up-regulation of FGF2 (encoded by the *FGF2* gene) was a pronounced predictor of paclitaxel resistance in serous ovarian cancer [40,41]. C-C motif chemokine ligand (CCL) and C-X-C motif chemokine ligand (CXCL) chemokines were associated with cancer immune evasion, and preoperative levels of these chemokines differ between cancer patients. Elevated levels of circulating CXCL4 + CCL20 + CXCL1 in combination can discriminate among ovarian cancer patients those who are predisposed to shorter progression-free survival and overall survival [42,43]. CXCL1 and CXCL8 were identified as being distinctive ovarian cancer markers [44,45]. The *IL6* gene (coding for interleukin-s, IL-6) was identified as the most up-regulated gene after post-neoadjuvant chemotherapy. Inordinately elevated IL-6 may drive, via the IL6/IER3 signaling axis, chemoresistance and disease recurrence of ovarian tumors; higher levels of IL-6 and VEGA-A were significantly associated with shorter progression-free survival [46,47,48]. It is also noteworthy that PSMB9 expression is up-regulated by GCS overexpression in ovarian cancer, and is associated with cancer resistance to platinum-based chemotherapy (Figure 5A) [35]. The *PSMB9* gene encodes proteasome subunit beta type-9 (PSMB9, also known as 20S proteasome subunit beta-1i), which possesses “trypsin-like” activity and is a component of the immunoproteasome involved in the processing of numerous MHC class-1 restricted T-cell epitopes [49]. PSMB9 epigenetically mediates immune response, including involvement of CD8+ T-cells. Overexpression of PSMB9 is a predictor of recurrence and, possibly, treatment response failure for high-grade serous epithelial ovarian carcinomas [50,51,52]. The *CLU* gene encodes clusterin (CLU, also known as apolipoprotein J), a cytoprotective chaperone protein associated with the clearance of cellular debris and with apoptosis [53]. CLU is a molecular chaperone responsible for aiding proper folding of secreted proteins, and its three isoforms (nuclear, cytosolic, and secretory) have been differentially implicated in pro-apoptotic (nuclear CLU) or anti-apoptotic processes (cytosolic and secretory isoforms) [54]. CLU is generally recognized as an anti-apoptotic molecule and a basis for drug resistance. CLU is a driving force of tumorigenesis or overexpressed after chemotherapy in ovarian cancer; however, its roles remain controversial for some cancer cells [55,56,57,58]. We found CLU expression to be down-regulated by GCS in NCI/ADR-Res cells that carry a deletion mutation in p53 exon-5 (Figure 5A) [26,28]. We also observed that GCS down-regulated the expression of MAPK8 with platinum-resistance (Figure 5A). Mitogen-activated protein kinase 8 (MAPK8, also known as JNK1) is a ubiquitous enzyme encoded by the *MAPK8* gene. In response to various cell stimuli, activated MAPK8 mediates immediate/early gene expression via targeting specific transcription factors. Activation of MAPK8 is required for TNF-α or radiation-induced apoptosis; stress-induced ceramide increase, and incipient apoptosis, depends on the activation of MAPK8 [59,60].

**Figure 6:**
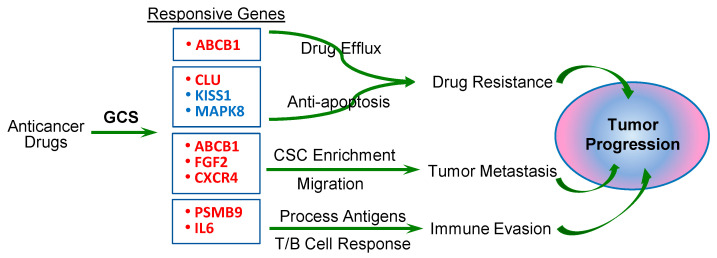
GCS modulates cell signaling pathways promoting drug resistance and metastasis in tumor progression. Under chemotherapy, anticancer drugs induce GCS overexpression and enhance ceramide glycosylation. Further, altered expression of GCS-responsive genes modulates at least four major cell pathways: (1) increase in drug efflux in cancer cells by the pump protein ABCB1 (also known as MDR1); (2) decrease in apoptosis and enhancement of proliferation of cancer cells by CLU, KISS1 MAPK8 (genes shown in light red indicate increased expression, and in light blue indicate decreased expression); (3) enrichment of cancer stem cells (CSCs) and enhancement of cell migration and drug resistance by ABCB1, FGF2, and CXCR4 in tumor metastasis; (4) mediation of the processing and presentation of antigens and MHC class-1 molecules, and immune cell response by a immunoproteasome PSMB9 and IL6 for immune evasion of tumor.

Drug resistance decreases therapeutic efficacy of anticancer drugs and enhances cancer survival and aggressive progression. Drug-resistant ovarian cancer often displays aggressive metastability and recurrence, most likely due to enrichment with cancer stem cells [61]. GCS plays a crucial role in mediating the stemness of cancer stem cells [9,10,62]. ALDH1 is commonly used as a marker of ovarian cancer stem cells, and ALDH1A3 and FGF2 within the microenvironment preserve the stemness of cancer stem cells [63,64,65,66]. The CD74, CXCL1, and IL-6 released from surrounding cancer-associated mesenchymal stromal cells (CA-MSCs) have been reported to increase cancer stem cells of ovarian cancers [67,68,69]. ALDH1A3, FGF2, and IL-6 have emerged as therapeutic targets for ovarian cancer [66]. As schemed in Figure 6, GCS up-regulates expression of GCS-responsive genes and modulates cell signaling pathways to enhance drug efflux, decrease apoptosis, and enrich cancer stem cells and even immune evasion, thus advancing drug resistance and tumor metastasis in tumor progression.

With responsiveness to stress, ceramide glycosylation catalyzed by GCS is indeed involved in regulating gene expression of cancer cells [8,70]. However, it has remained unclear how GCS mediates the expression of GCS-responsive genes in cancer cells. Based on available literature evidence, it is highly possible (cf. Figure 5B), that the increased levels of glycosphingolipids (GSLs) following ceramide glycosylation, which is the first limiting reaction in GSL synthesis, promotes protein kinase function (*cSrc* family members, EGFR) in GSL-enriched microdomains (GEM) in plasma membranes, in turn activating β-catenin/T-cell factor (TCF)-mediated transcription and other transcription factors so as to up-regulate expression of GCS-responsive genes (ABCB1, FGF2, IL6, etc.). The aggregate alterations may even contribute to pronounced expression of missense p53 mutants via deleterious RNA methylation and pre-mRNA splicing [6,8,9,16,71,72,73,74]. Our previous works and others elucidated that GCS modulates the β-catenin signaling pathway to up-regulate the expression of ABCB1, FGF2, and METTL3 in cancer cells [6,8,9,16]. Furthermore, decreased levels of ceramide and other sphingolipids, resulting from increased ceramide glycosylation, can reduce or abolish ceramide-based signaling (ceramide kinase, MAPK8/JNK1, PI_3_K, PKC). These protein kinases otherwise act to constrain deleterious overexpression of particular genes (e.g., *IL6*), and even that of cancer p53 mutants (deletion in exon-5) and isoforms of chemokine ligands via downstream transcription factors [75,76,77,78,79]. Altogether, our present study systemically identified GCS-responsive genes prominently responsible for cancer drug resistance and aggressive metastasis. These results can help us understand how GCS-catalyzed ceramide glycosylation mediates cross-talk between sphingolipid alterations and cancer cell responses to stress, and further underscores the crucial role played by GCS in cancer progression. GCS-responsive genes are key players in cancer drug resistance and metastasis. They might serve as biomarkers for monitoring therapeutic response and therapeutic targets for developing new therapy.

## 4. Materials and Methods

### 4.1. Cell Lines and Cell Culture

Drug-resistant human NCI/ADR-RES ovary adenocarcinoma cells [80] were kindly provided by Dr. Kenneth Cowan (UNMC Eppley Cancer Center, Omaha, NE, USA) and Dr. Merrill Goldsmith (National Cancer Institute, Bethesda, MD, USA) [81]. ADR-RES/GCS and ADR-RES/asGCS are sublines of NCI/ADR-RES generated from clone selections with geneticin (G418, 400 μg/mL) after transfection of parental cells with pcDNA 3.1-asGCS (GCS antisense) or pcDNA 3.1-GCS (10 μg/mL, 100 mm dish) by co-precipitation with calcium phosphate (Mammalian Transfection Kit; Stratagene, La Jolla, CA, USA) [25]. pcDNA 3.1/his A plasmid was used in control transfections to generate ADR-RES/mock cells. Human ovarian cancer A2780 cells were kindly provided by Dr. M. Hollingshead (Division of Cancer Treatment and Diagnosis Tumor Repository at the National Cancer Institute). A2780 and NCI/ADR-RES cells were cultured in RPMI-1640 medium containing 10% fetal bovine serum (FBS), 100 units/mL penicillin, 100 µg/mL streptomycin, and 584 mg/liter l-glutamine. Cells were maintained in an incubator humidified with 95% air and 5% CO_2_ at 37 °C. Cells were passaged every 2–3 days before they reached 90% confluency for no more than 12 passages. Cell lines were authenticated in November 2022 at the John Hopkins University Fragment Analysis Facility (Baltimore, MD, USA) using an Applied Biosystems Identifiler System to test for 16 STR markers and amelogenin for gender determination. Authenticity was confirmed against the ATCC database (https://bioinformatics.hsanmartino.it/clima2/, accessed on 28 November 2022) and the NCI-60 database published [82]. Dactinomycin (DAC, also named actinomycin D) was purchased from Sigma-Aldrich (St. Louis, MO, USA), and other reagents for cell cultures were purchased from Thermo Fisher Scientific (Dallas, TX, USA).

### 4.2. RT-PCR Analysis of p53 mRNA

This assessment was performed as described previously [83]. Briefly, total RNA and mRNA were extracted and purified using an SV total RNA isolation kit and PolyATract mRNA isolation system (Promega, Madison, WI, USA). Equal amounts of RNA (500 ng) were used to synthesize first-strand DNA using the SuperScriptR III kit (Invitrogen, Carlsbad, CA, USA). Five microliters of first-strand DNA from each sample was amplified using the Platinum^®^ Blue PCR SuperMix kit (Invitrogen). Pairs of primers listed in Table 1 were used in PCR amplification. Glyceraldehyde-3-phosphate dehydrogenase (GAPDH), an endogenous control (200 bp), was amplified using upstream primer 5′-ATGGGGAAGGTGAAGGTCGG-3′ and downstream primer 5′-TCCACCACCCTGTTGCTGTA-3′. The PCR amplification was performed in 35 cycles of denaturation at 94 °C for 30 s, annealing at 55 °C for 30 s, and extension at 72 °C for 60 s.

### 4.3. DNA Fragmentation and Apoptosis Assay

Induced apoptosis and DNA fragmentation analyses were performed as described previously [2,25]. Briefly, cells of ADR-RES/GCS, ADR-RES/asGCS, or ADR-RES/mock lines (0.5 × 10^6^ cells) were seeded in 10 cm dishes in medium containing 5% FBS. After attachment, cells were treated with 25 nM dactinomycin (DAC) for 48 h. After harvest by trypsin-EDTA and centrifugation, cells were digested with lysis buffer (10 mM Tris-HCl, pH 8.0, 100 mM NaCl, 25 mM EDTA, 0.5% SDS, 0.3 mg/mL proteinase K). DNA was extracted with phenol/chloroform/isoamyl alcohol (25:24:1, *v*/*v*/*v*) and precipitated by incubating in one-half volume of 7.5 M ammonium acetate plus two volumes of 100% ethanol at −20 °C overnight, followed by centrifugation (10,000× *g*, 20 min, 4 °C). Contaminating RNA was digested in RNA digestion buffer (10 mM Tris-HCl, 0.1 mM EDTA, 0.1% SDS, 100 U/mL RNase mixture). Re-extracted DNA (2–10 μg) was separated by electrophoresis on a 2% agarose gel in TAE buffer (40 mM Tris-acetate, 1 mM EDTA, pH 8.3) and visualized with ethidium bromide under UV light.

### 4.4. Western Blot Analysis

Western blotting was carried out as described previously [9,75]. Briefly, cells or tissue homogenates were lysed in NP40 cell lysis buffer (Biosource, Camarillo, CA, USA) to extract total cellular proteins once treatment was concluded. Total protein content was assessed via a bicinchoninic acid (BCA) protein assay kit (Pierce, Rockford, IL, USA). Equal amounts of proteins (50 µg/lane) were resolved using 4–20% gradient SDS-PAGE (Life Technology, Waltham, MA, USA). After transferring, blots of nitrocellulose membrane were blocked in 5% fat-free milk in 0.05% Tween-20, 20 mM phosphate-buffered saline, pH 7.4 (PBST), and then incubated with each one of the primary antibodies (1:500 or 1:5000 dilution) at 4 °C overnight. After PBS washing, these blots were incubated with corresponding horseradish peroxidase-conjugated secondary antibodies (1:5000 dilutions) and developed using SuperSignal West Femto substrate (Thermo Fisher Scientific). Glyceraldehyde-3-phosphate dehydrogenase (GAPDH) was used as a loading control for cellular protein. Relative protein levels present were calculated from the OD values, normalized against those for GAPDH. Antibody against p53 phosphorylated at Ser15 was purchased from Cell Signaling Technology (Danvers, MA, USA). Antibodies for mouse IFN-γ, p53, and GAPDH were purchased from Santa Cruz Biotechnology (Dallas, TX, USA).

### 4.5. Microarray Analysis

RNA samples of cell lines were extracted using an SV total RNA isolation kit (Promega, Madison, WI, USA) and the quality of RNA was assessed by using Bioanalyzer with an Agilent RNA 6000 Nano kit (Agilent, Santa Clara, CA, USA) for RNA integrity [84,85,86]. Probe synthesis and array hybridization were performed using established Affymetrix methods [87,88]. Briefly, 2 μg purified RNA was reverse-transcribed to cDNA using T7 promoter-(deoxythymidine)24 primer. After second-strand synthesis, biotin-labeled cRNA was generated from the double-strand template using T7 RNA polymerase. The quality of the cRNA probe was verified by running an aliquot on agarose gel. Exactly 20 μg of labeled cRNA was hybridized onto an Affymetrix GeneChip^®^ Human Genome U133 Plus 2.0 chip for 16 h at 45 °C in 300 mL premixed hybridization solution containing labeled hybridization control prokaryotic genes (bioB, bioC, bioD, and cre). Replicate spots for each control gene are present on the chip. Chips were washed in the GeneChip Fluidics Station automatic washer and scanned on the GeneArray^®^ fluorometric scanner 3000 running Gene-Chip Operating Software 1.2 to generate gene expression data (CEL files). The GeneChip^TM^ Human Genome U133 Plus 2.0 Array (Affymetrix; catalog 900466) was purchased from Thermo Fisher Scientific (Waltham, MA, USA).

### 4.6. Database Submission of Microarray Data and Further Analysis

The microarray data were deposited in the Gene Expression Omnibus (GEO) database: http://www.ncbi.nlm.nih.gov/geo/ (accessed on 13 June 2024). The GEO accession number for the platform is GSE42392, samples GSM1038716–GSM 1038775. The data from the Affymetrix microarray were further analyzed by Transcriptome Analysis Console (TAC) v4.0 Software (http://www.affymetrix.com/support/technical/byproduct.affx?product=tac; accessed on 13 June 2024). Further bioinformatics analysis for the visualization, interpretation, and pathways analysis were obtained via the REACTOME server (https://reactome.org/; accessed on 13 June 2024).

### 4.7. Data Analysis

All experiments in cell models were repeated twice more. Data are expressed as mean ± SD. Two-tailed Student’s *t* tests and one-way ANOVA tests were used to compare the continuous variables between groups, using the Prism v9 suite (GraphPad, San Diego, CA, USA). All *p*-values equal to or less than 0.01 comparisons were regarded as statistically significant.

## 5. Conclusions

Silencing GCS expression sensitized drug-induced apoptosis in NCI/ADR-RES ovarian cancer cells, which commonly express high levels of GCS and p53 mutant. Gene expression profiling of cell models characterized 41 GCS-responsive genes that are associated with cancer progression. Integrated genomic analysis of ovarian cancer cases further indicates GCS with eight of these GCS-responsive genes attributes under chemotherapy. Ceramide glycosylation by GCS results in cancer drug resistance via up-regulating the GCS-responsive genes perpetrating tumor progression.

## Figures and Tables

**Table 1:** PCR primers for p53 mRNA and their products.

Primer	Sequence	Product (bp)	Positions
del-p53 (exon-5) forward reverse	5′-TCACTGCCATGGAGGAG-3′ 5′-TTGAGGGCAGGGGAG-3′	400	113–512
Pan-p53 forward reverse	5′-TTGCCGTCCCAAGCAATG-3′ 5′-AAGTCACAGACTTGGCTGTCCCAGA-3′	268	223–490

del-p53 is an 18 bp deletion in human p53 mRNA (ORF 496–513, accession number BC003596) found in NCI/ADR-RES cells.

**Table 2:** UGCG (GCS) modulated expression of genes in NCI/ADR/RES ovarian cancer cells with dactinomycin treatments. Among differentiated expression levels of 41 genes, GCS transfection up-regulated 22 genes (light red, >2-fold increase in ADR-RES-GCS cells transfected with GCS and <2-fold in ADR-RES/asGCS cells transfected with asGCS) and down-regulated 19 genes (light blue, <2-fold decrease in ADR-RES/asGCS cells and >2-fold in ADR-RES/GCS cells) while asGCS transfection achieved the opposite in NCI/ADR-RES cells. A negative fold change (signed linear fold change) indicates down-regulation.

Gene Symbol	Description	GCS vs. Mock (Fold)	asGCS vs. Mock (Fold)
CXCL8	chemokine (C-X-C motif) ligand 8	6.9	−2.5
PSMB9	proteasome subunit beta 9	5.5	−2
CXCL1	chemokine (C-X-C motif) ligand 1	5.3	−2.8
INHBA	inhibin beta A	4.6	−3.3
CCL20	chemokine (C-C motif) ligand 20	4.4	−2.9
APOE	apolipoprotein E	3.6	−2.3
ABCB1	ATP-binding cassette subfamily B member 1	3.2	−5.5
FGF2	fibroblast growth factor 2 (basic)	3.1	−21.9
PMEPA1	prostate transmembrane protein, androgen induced 1	3	−2.4
CD74	CD74 molecule	2.8	−2.4
IL6	interleukin 6	2.7	−3.3
MFSD6	major facilitator superfamily domain containing 6	2.6	−2.1
LAMC2	laminin, gamma 2	2.5	−2.5
C1S	complement component 1, s subcomponent	2.4	−3.3
APLP2	amyloid beta (A4) precursor-like protein 2	2.3	−2.1
SNX19	sorting nexin 19	2.3	−2.5
ZNF568	zinc finger protein 568	2.2	−2.1
NFKBIZ	nuclear factor of kappa light polypeptide gene enhancer in B-cells inhibitor, zeta	2.2	−3.5
OSR2	odd-skipped related transciption factor 2	2.2	−2
KISS1	KiSS-1 metastasis-suppressor	2.1	−2.2
ALDH1A3	aldehyde dehydrogenase 1 family, member A3	2.1	−3.9
TMCC1	transmembrane and coiled-coil domain family 1	2	−2.8
UGCG (GCS)	UDP-glucose ceramide glucosyltransferase	1.3	−1.1
GALC	galactosylceramidase	−29.6	−17.4
RIOK3	RIO kinase 3	−2.1	2.1
ARRDC4	arrestin domain containing 4	−2.1	3.3
TAF1D	TATA box binding protein associated factor 1D	−2.1	2
MAPK8	mitogen-activated protein kinase 8	−2.1	2.1
LYRM1	LYR motif containing 1	−2.2	2.2
ZNF177	zinc finger protein 177	−2.3	2.2
TTC28-AS1	TTC28 antisense RNA 1	−2.4	3.1
CNRIP1	cannabinoid receptor interacting protein 1	−2.4	2
NAP1L5	nucleosome assembly protein 1-like 5	−2.5	2.6
TEX19	testis expressed 19	−2.5	2.1
RARB	retinoic acid receptor, beta	−2.5	3.4
LINC00662	long intergenic non-protein coding RNA 662	−2.5	3.8
CXCR4	chemokine (C-X-C motif) receptor 4	−2.6	2.2
PAG1	phosphoprotein membrane anchor with glycosphingolipid microdomains 1	−2.7	2.4
CLU	clusterin	−2.8	2.9
TMEFF2	transmembrane protein with EGF-like and two follistatin-like domains 2	−2.8	2.3
MFAP2	microfibrillar associated protein 2	−2.9	2.8
OVOS2	ovostatin 2	−3.9	2.1
SFTA1P	surfactant associated 1, pseudogene	−4.6	2.1

**Table 3:** GCS-Responsive Genes in Ovarian Cancer. Data collected from National Cancer Institute GDC Data Portal (https://portal.gdc.cancer.gov/, accessed on 13 June 2024). A negative fold change (signed linear fold change) indicates down-regulation. NS indicates non-significant.

Gene Symbol	Tumor (n = 426)	Normal (n = 88)	Fold	Adj *p*-Value
CXCL8	5.95	0.86	3.7	2.01 × 10^−18^
PSMB9	71.24	17.55	3.9	6.24 × 10^−23^
CXCL1	6.43	0.57	4.7	4.47 × 10^−20^
MT1M	2.73	5.89	−1.9	NS
INHBA	2.23	1.79	1.2	NS
CCL20	4.66	0.89	5.2	3.10 × 10^−29^
APOE	260.91	363.11	−1.4	NS
ABCB1	0.55	4.18	−3.3	5.33 × 10^−97^
FGF2	0.22	7.53	−7.0	6.29 × 10^−187^
PMEPA1	15.14	10.91	1.4	NS
CD74	2309.00	189.54	12.1	5.89 × 10^−52^
IL6	9.72	34.96	−3.4	4.03 × 10^−55^
MFSD6	12.42	4.34	2.5	1.56 × 10^−29^
LAMC2	23.77	0.88	13.2	4.23 × 10^−83^
C1S	77.70	945.41	−12.0	4.61 × 10^−73^
APLP2	168.08	154.48	1.0	NS
SNX19	16.92	21.60	−1.3	NS
LOC643201				NS
NFKBIZ	4.31	24.02	−4.7	1.92 × 10^−45^
OSR2	5.66	80.88	−12.3	1.58 × 10^−77^
ZNF568	4.03	6.07	−1.4	NS
ALDH1A3	2.30	2.84	−1.2	NS
IFIT1	40.64	8.15	4.6	1.96 × 10^−24^
KISS1	0.43	0.00	1.4	NS
TMCC1	11.42	8.98	1.3	NS
UGCG (GCS)	9.40	9.91	−1.0	NS
AKT3	1.80	27.46	−10.2	5.57 × 10^−86^
ARRDC4	2.37	5.44	−1.9	NS
CDK6	1.95	2.25	−1.1	NS
MAPK8	14.71	21.55	−1.4	NS
RIOK3	20.05	30.89	−1.5	NS
TAF1D	61.50	113.83	−1.8	NS
LYRM1	31.50	33.43	−1.1	NS
IGFBP3	73.80	163.27	−2.2	3.31 × 10^−12^
ZNF177	1.66	7.52	−3.2	1.05 × 10^−51^
CNRIP1	2.58	43.46	−12.4	7.14 × 10^−125^
TTC28-AS1	9.09	32.05	−3.3	1.37 × 10^−58^
LINC00662	9.65	8.12	1.2	NS
NAP1L5	1.39	6.78	−3.3	2.06 × 10^−67^
RARB	2.12	5.89	−2.2	2.37 × 10^−20^
TEX19	0.04	0.00	1.0	NS
CXCR4	65.44	5.78	9.7	2.42 × 10^−63^
PAG1	3.63	1.17	2.1	6.33 × 10^−22^
CLU	1874.12	671.15	2.8	1.43 × 10^−6^
TMEFF2	0.02	0.06	−1.0	NS
MFAP2	98.05	42.01	2.3	4.58 × 10^−5^
TFRC	30.48	67.49	−2.2	5.51 × 10^−15^
OVOS2	1.59	1.02	1.3	NS
GJA1	19.68	28.36	−1.4	NS
SFTA1P	0.27	0.34	−1.1	NS
PLEKHA8	1.82	3.83	−1.7	NS
PDAP1	81.42	85.53	−1.1	NS
MATR3	6.00	6.62	−1.1	NS
TRIM59	5.41	1.00	3.2	2.43 × 10^−51^
ARMCX4	2.48	35.09	−10.3	8.83 × 10^−151^
MALAT1	577.01	714.13	−2.2	NS
GALC	6.88	15.41	1.3	4.89 × 10^−27^

## Data Availability

The datasets generated and/or analyzed during the present study are available from the corresponding author upon reasonable request.
